# TD-DFT Simulation and Experimental Studies of a Mirrorless Lasing of Poly[(9,9-dioctylfluorenyl-2,7-diyl)-co-(1,4-diphenylene-vinylene-2-methoxy-5-{2-ethylhexyloxy}-benzene)]

**DOI:** 10.3390/polym13091430

**Published:** 2021-04-29

**Authors:** Mamduh J. Aljaafreh, Saradh Prasad, Mohamad S. AlSalhi, Raya H. Alhandel, Reem A. Alsaigh

**Affiliations:** 1 Department of Physics and Astronomy, College of Science, King Saud University, Riyadh 11451, Saudi Arabia; maljaafreh@ksu.edu.sa (M.J.A.); srajendra@ksu.edu.sa (S.P.); rhalhandhal@gmail.com (R.H.A.); rsaigh@ksu.edu.sa (R.A.A.); 2 Research Chair on Laser Diagnosis of Cancers, Department of Physics and Astronomy, College of Science, King Saud University, Riyadh 11451, Saudi Arabia

**Keywords:** CP PFO-co-PPV-MEHB, sub-nanosecond TRS, amplified spontaneous emission (ASE) spectra, green emitter

## Abstract

In this work, we investigate the TD-DFT simulation, optical, and mirrorless laser properties of conjugated polymer (CP) Poly[(9,9-dioctylfluorenyl-2,7-diyl)-co-(1,4-diphenylene-vinylene-2-methoxy-5-{2-ethylhexyloxy}-benzene)], also known as (PFO-co-PPV-MEHB) or ADS125GE. TD-DFT calculations were performed for three monomer units with truncated tails using time-dependent density functional theory (TD-DFT) calculations. The calculations showed a highest occupied and lowest unoccupied molecular orbital (HOMO-LUMO) structure and a very high oscillator strength of 6.434 for the singlet-singlet transition at 374.43 nm. Experimentally, the absorption and fluorescence spectra were examined at various concentrations in verity of solvents, such as benzene, toluene, and hexane. The experimental results obtained in hexane were comparable with theoretical UV-VIS spectra calculated under vacuum. Amplified spontaneous emission (ASE) spectra peaked at approximately 509 nm for CO PFO-co-PPV-MEHB in solution and were obtained at suitable concentrations and pump energies. Additionally, the photochemical stability of this CP and coumarin (C510) were compared. Time-resolved spectroscopy (TRS) studies with a sub-nanosecond resolution were performed for the CO under various pump energies. These results showed the excited state dynamics and single-pass optical gain of CO PFO-co-PPV-MEHB.

## 1. Introduction

Since their discovery [1,2], four decades of research has been performed on conjugated materials (polymers and oligomers) due to their captivating photophysical properties. The properties of these special materials, such as high quantum yield, high chromophore density, large Stokes shift, wavelength tunability, and large optical gain, have been studied in depth [3]. These extraordinary properties make these polymers ideal in many applications, such as in laser-active media [4,5,6,7,8] flexible FET (field-effect transistors) [9], photovoltaic devices [10], and photodiodes, as well as light-emitting diodes (LEDs) [9,10,11]. Both conjugated polymers (CPs) and conjugated oligomers (COs) are appealing laser materials and can produce lasing at proper concentrations in solid-state, thin-film, and liquid forms [12,13,14,15,16]. ASE is produced when the active medium is optically excited by an intense laser source. Moreover, the ASE feedback can be converted to a laser when an optical cavity is combined with the system [17]. In general, materials that can achieve ASE can produce lasing under an optical cavity. However, some materials produce lasing in the cavity but cannot produce ASE without a cavity.

Many CPs exhibit amplified spontaneous emission (ASE), mirrorless lasing with high intensity, spatial coherence, and low temporal coherence. CPs as active gain media have produced ASE and lasing, which could be tunable over a wide range in the visible spectrum [18,19,20]. Broadband ASE is used as a light source, which benefits many applications such as fiber sensing and telecommunications. These polymers are highly efficient in energy transfer to other polymers, oligomers, and perovskite quantum dots.

The first report on ASE from CPs was presented by Moses et al. MEH-PPV produced ASE via optical pumping in solution and film. The quantum efficiency of MEH-PPV was compared to that of the conventional dye rhodamine 6G [21]. Another group reported laser emission from TOP-PPV in solution [22]. Another CP, poly(9,9-dioctylfluorene) (PFO), was also well studied and found to produce ASE at different wavelengths depending on the concentration in solution. Thin films of PFO produce ASE from the β-phase.

In 2007, Redmond’s team studied the first incidence of single-nanowire lasing under optical pumping for CO PFO, in which nanowires were formed through the template wetting method and exhibited a single Fabry–Pérot mode [23]. The threshold energy was 100 nJ. Moreover, the M. S. AlSalhi group investigated the properties of a CP as an active laser medium and proved the presence of excimeric and dimeric states of the CP. They studied the spectral temporal profile as well as ASE from the CP under different concentrations, solvent types, temperatures, and laser energy excitations using a Princeton Instruments PI-MAX 4 ultrafast-gated emCCD with an Acton picosecond spectrograph [24]. R. H. Friend and his team organized an intensive research work on CP blend laser systems, especially with the Forster energy transfer (FRET) mechanism [25,26].

Copolymerization is a technique that enables existing monomers to be combined to achieve new bandgaps and emission properties. The ASE properties of copolymer PFO-co-pX were studied by S.A Alfahd et al. There have only been minimal studies carried out on copolymers with a combination of PFO and PPV derivatives.

However, in general, DFT simulation, optical, and laser studies of conjugated polymers are rare [27,28,29,30,31] and in particular, none are about PFO-co-PPV-MEHB. To the best of our knowledge, this work could be the first work on the TD-DFT calculation of CP PFO-co-PPV-MEHB. We calculated electronic properties such as structure optimization; the E_HOMO_, E_LUMO_, E_Gap_; the dipole moment; and the oscillator strength of PFO-co-PPV-MEHB for structures optimized under the three-monomer truncated-tail oligomer model using the DFT/Coulomb-attenuated method at the B3LYP (CAM-B3LYP)/6-31G(d,p) level by Gaussian 16 and other software. Furthermore, experimental studies of PFO-co-PPV-MEHB spectral and mirrorless laser properties in toluene under transverse excitation were performed. The pump source was the Nd: YAG laser of 355 nm. We demonstrate that under a suitable concentration and a low pump energy, CO PFO-co-PPV-MEHB can produce ASE at 508 in toluene. TRS studies display the ASE in 3D features, with the wavelength, spectral amplitude, and time as the X-, Y-, and Z-axes, correspondingly.

## 2. Materials and Methods

The molecular structure of CP PFO-co-PPV-MEHB could be explained as a group of bridged structures. The molecule (A) 1-methyl-4-[(1E)-prop-1-en-1-yl] benzene shown in Seg. A is connected to both sides of the familiar CP poly[(9,9-dioctylfluorenyl-2,7-diyl)] (PFO) shown in Seg. B. Molecular segment A is connected to another polymer (1,4-diphenylene-vinylene-2-methoxy-5-{2-ethylhexyloxy}-benzene) resembling the MEH-PPV structure, as shown in Seg. C. The cumulative structure becomes [(A)-(B)-(A)-(C)] n. This approach is taken to modify the HOMO-LUMO gap. The calculated dipole moment is 1.4334525 Debye for n = 3, which is for the structure optimized using the DFT/Coulomb-attenuating method (Becke-3 Parameter-Lee-Yang-Parr) CAM-B3LYP/6-31G(d,p) basis set and the augmented structure is shown in Supplementary Figure S1. We used a workstation that consisted of an Intel i7 processor, 64 GB RAM, and 1 TB SSD hard disk for all our simulations.

The CP has 65,000 ± 35,000 g mol^−1^ as its molecular weight (Mw). American Dye Source, Inc. (Baie-D’Urfé, Montreal, QC, Canada) provided the CP material. (ADS125GE) The Coumarin 510 has an Mw of 318.4 g/mol and was precured from Exciton Luxottica. A Perkin Elmer Lambda 950 spectrophotometer (Llantrisant, UK) was utilized to record the absorption spectra. The fluorescence spectra at an excitation wavelength of 355 nm from a xenon flash lamp were investigated utilizing a spectrofluorometer (LS 55, Llantrisant, UK). To achieve transverse pumping, the tripled frequency (355 nm) of a 5 ns Nd:YAG laser was focused by a quartz cylindrical lens (5 cm focal length). The cylindrical lens focused the pulse onto a horizontal strip of 1 cm × 2 mm (length × width). One fiber was linked to an Ocean Optics spectrograph (Maybachstrasse, Ostfildern, Germany) to record the spectra. Another fiber was linked to a Princeton Instruments PI-MAX 4 ultrafast-gated emICCD, which had a gate delay of less than 500 ps. The camera had a high-speed electrically controlled shutter that allowed events to be recorded with a sub-nanosecond time resolutions (please see [32]).

**Figure 1:**
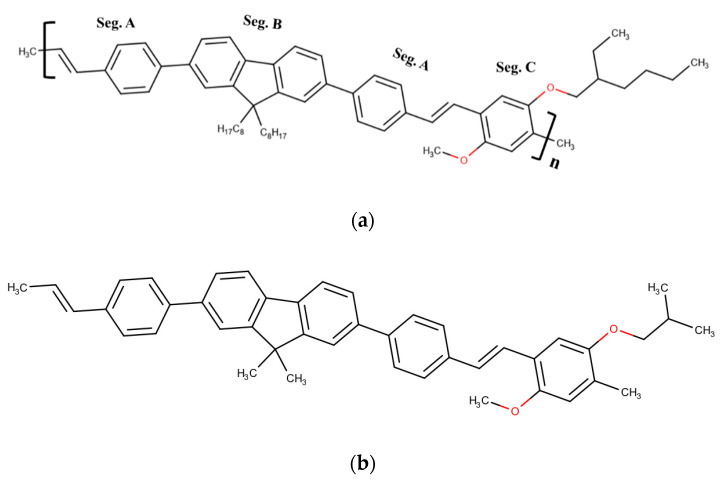
(**a**) Molecular structure of CP poly[(9,9-dioctylfluorenyl-2,7-diyl)-co-(1,4-diphenylene-vinylene-2-methoxy-5-{2-ethylhexyloxy}-benzene)] (i.e., PFO-co-PPV-MEHB). (**b**) Tail-truncated molecular structure utilized for the DFT calculation of PFO-co-PPV-MEHB.

The monomer of PF-co-MEH-PPV is very large and requires significant computational resources. The full-scale simulation of the complete polymer structure with many monomers could be challenging. However, an oligomer structure with three connected monomers is accepted in practice for simulating polymer properties; hence, we adopted the n = 3 oligomer approach. To further reduce the computational burden, we cut short the tail of PFO and MEH-PPV segments, as shown in Figure 1b, since its contribution to optical properties is insignificant. However, practically, the long tail offers a higher solubility in many common solvents [33]. The TD-DFT calculations were performed via the following steps. The molecular structure was created using the Gaussian View 6 software. The structure was optimized with Gaussian 16 using the DFT/CAM-B3LYP/6-31G(d,p) basis set [34]. Next, the UV-VIS spectra, HOMO–LUMO structure, and dipole moment were calculated using the CAM-B3LYP/6-31G(d,p) basis set. Other calculations, such as acceptor-donor sites, polarizability, and charge distribution, were calculated using molecular dynamics (HFF) in the MarvinSketch software, as shown in Figure S2a–c. The charge distribution showed that the central ring structure was negative, more negative charges were found at oxygen sites and the edge of the ring was mostly positive. The CP polarizability was calculated for n = 3 was 229.15 ${Å}^{3}$ , which shows that CP has a high polarizability for external electric fields, such as optical pumping. The H bond acceptor and donor site were calculated and it was found that each monomer contained 2 acceptor and 4 acceptor sites at oxygen; hence, the CPs have more solubility in a medium polar solvent.

## 3. Results and Discussion

### 3.1. DFT Calculation

**Figure 2:**
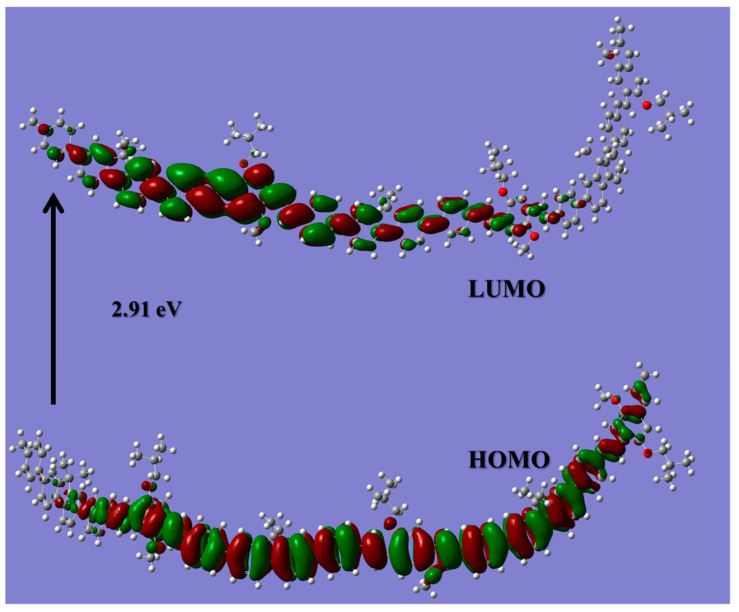
Frontier molecular orbital (HOMO-LUMO) structure of PFO-co-PPV-MEHB (tail-truncated and n = 3 model) calculated using the CAM-B3LYP/6-31G(d,p) basis set.

CPs consist of a large number of atoms; hence, the simulation of CPs is considered to be challenging. Thus, many studies have adopted an oligomeric model of the compound [35,36,37]. The absorption spectra and energy gap of the PF-co-MEH-PPV were simulated with three monomer units (n = 3) based on the HOMO-LUMO energy gap calculations. Figure 2 shows the HOMO-LUMO structure of the three-monomer model. The HOMO (Frontier orbital) densities are aligned vertically in the major part of the ring structure; however, the density diminishes in the last monomer at one end (left). The LUMO densities are highly delocalized and align horizontally along the molecular backbone. The LUMO density is higher at the end of the oligomer model where the HOMO density is diminished. The movement of the orbital densities upon excitation significantly improves the optical and electronic properties of the CP. The calculated bandgap is 2.91 eV. The HOMO lies at −6.83 and the LUMO lies at −3.92 eV. In the same manner, the structural and optoelectronic properties of CPs and CO (PCDTBT) have been examined by DFT and TD-DFT [38].

**Figure 3:**
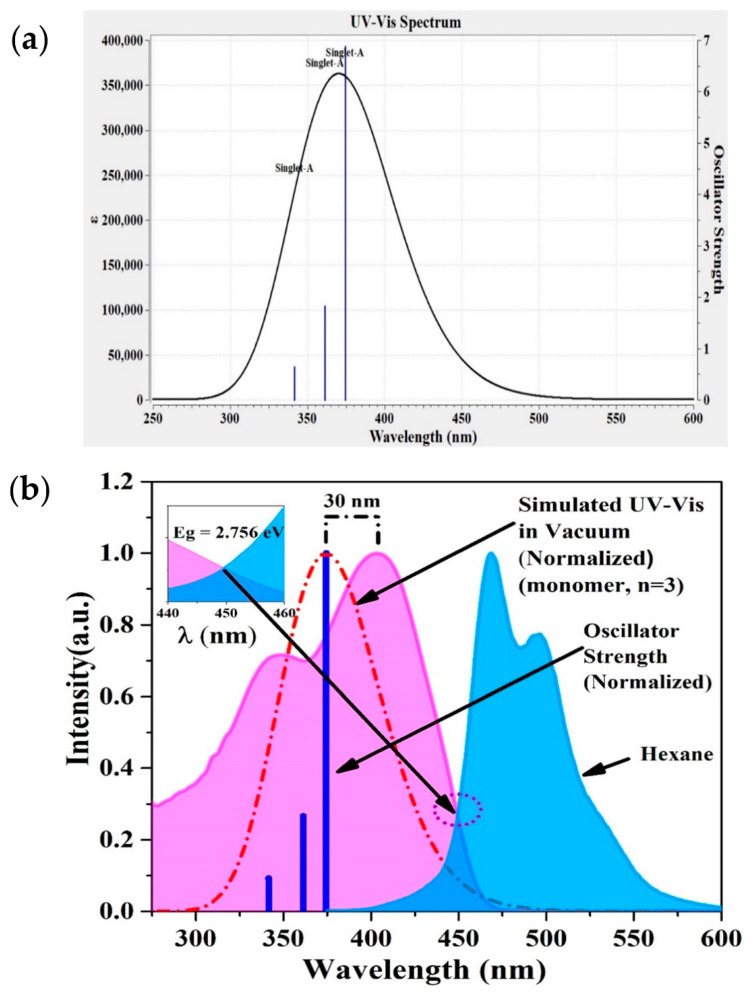
(**a**) UV-VIS and oscillator strength profile of PFO-co-PPV-MEHB (tail-truncated and n = 3 model). (**b**) Absorption of the low-concentration CP in hexane to identify the bandgap using the intersection principle and comparison with the stimulated spectrum.

The UV-VIS spectrum for PFO-co-PPV-MEHB (n = 3) in vacuum was simulated using the DFT/CAM-B3LYP/6-31G(d,p) basis set, as shown in Figure 3a. The results show three singlet oscillator strengths at wavelengths of 341.66, 361.38, and 374.51 nm with high values of f = 0.633, 1.8254, and 6.8762, respectively.

Hexane has a very low dielectric contestant and is a nonpolar solvent; hence, its effect on any molecule (solute) is much less than that of most solvents. Hence, it is optimal for comparison with the results simulated in vacuum. The difference between the simulated and experimental absorption spectra ( $\lambda_{\mathrm{Exp}}-\lambda_{\mathrm{sim}})$ is 30 nm, as presented in Figure 3b. This difference could be attributed to the solvent dielectric constant and concentration of the CP in hexane. However, the simulated singlet oscillator strengths matched the peaks and shoulder of the hexane absorption spectra.

Figure 3b shows that the bandgap calculated using the absorption and fluorescence intersection method is 2.756 eV. The purple dotted circle highlights the intersection of the absorption and fluorescence spectra, which is zoomed in on and shown in an inset of Figure 3b. This result is in good conformity with the simulated bandgap of 2.91 eV (in hexane). The disturbance could be due to the approximation of the polymer structure and repetition units (n = 3) and a change in the dielectric constant of the solvent. However, the experimental results show two distinctive bands that correlate with the simulated singlet oscillator strengths. Additionally, the experimental results contain three features, two peaks and a shoulder, which could be attributed to the three singlet oscillator strengths found using the simulation methods. The electronic circular dichroism (ECD) result is shown in Figure S3.

Figure S4 shows an estimation of the HOMO−LUMO gaps for PFO-co-PPV-MEHB using the bandgap extrapolation of oligomers (n = 1 to 5). The calculation was performed using the same TD-DFT method for all repetitive monomer units (n = 1 to 5), but the figures show only n = 3 for a clear presentation of the HOMO LUMO structure. The linear fitting gives the equation E_g_ (eV) = 2.7667 + 0.3408 × (1/n). When the value of n is large, the second term tends to become negligible. Thus, the calculated bandgap value of the polymer is 2.7667 eV using the extrapolation method. This value is comparable with the experimentally measured bandgap of 2.756 eV.

### 3.2. Absorption and Fluorescence Spectra of the CP in Toluene

**Figure 4:**
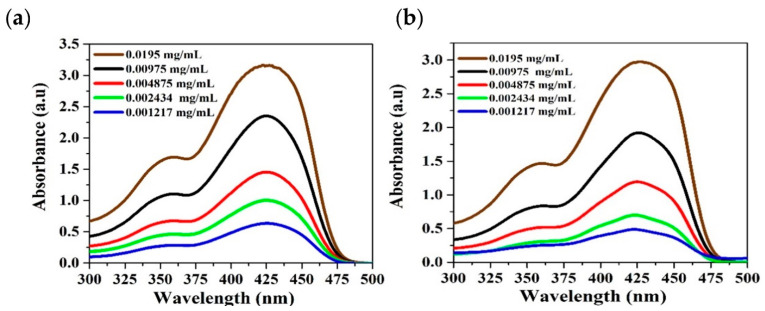
(**a**,**b**) Absorption spectra of PFO-co-PPV-MEHB in (**a**) toluene and (**b**) benzene at various concentrations.

Figure 4 presents the absorption spectra of the CP in toluene and benzene at different concentrations from 195 × 10^−4^–12.17 × 10^−4^ mg/mL. It was found that there are three diverse features. The first is a peak at approximately 355 nm and the second is the main peak at 425 nm, with the third being a shoulder at 370 nm. The optical density decreased from a high value to a low value as the concentration decreased. The absorption spectral profile remained the same, and the full width at half maximum (FWHM) of the maximum peak at 425 nm decreased with the decreasing PFO-co-PPV-MEHB concentration. The absorption spectra in benzene are very close to those of PFO-co-PPV-MEHB in toluene. Figure 4b shows the absorption features in benzene.

**Figure 5:**
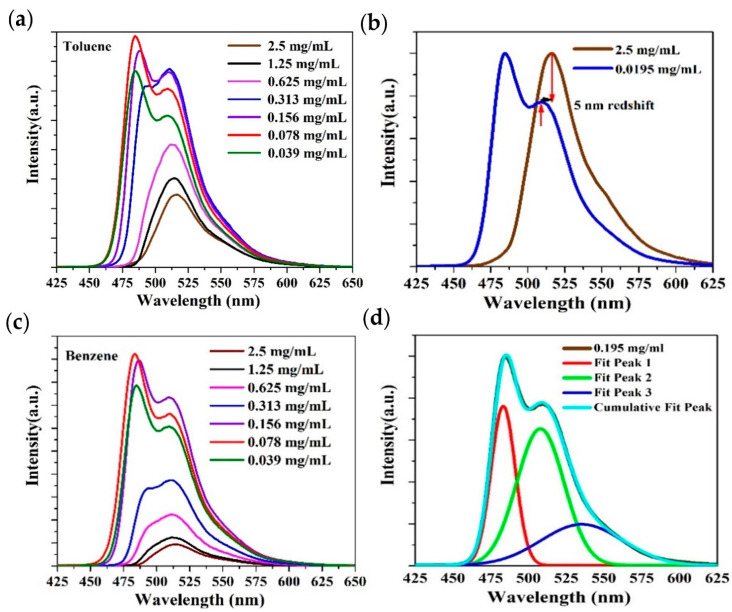
(**a**) Emission spectra of PFO-co-PPV-MEHB for various concentrations in toluene. (**b**) Redshift of the peak fluorescence spectra of PFO-co-PPV-MEHB in toluene. (**c**) Fluorescence spectra in benzene from high to low concentrations. (**d**) Deconvolution of the fluorescence spectrum of PFO-co-PPV-MEHB at 0.0195 mg/mL.

Figure 5a demonstrates the emission spectra of PFO-co-PPV-MEHB in toluene for different concentrations ranging from 2.5 mg/mL to 195 × 10^−4^ mg/mL. At low concentrations, fluorescence peaks occur at 485 nm and 510 nm, with a tail at 550 nm. Up to a concentration of 0.078 mg/mL, the fluorescence increases; after that, when the concentration (0.156 mg/mL) is further increased, the fluorescence intensity redshifts and the spectral profile shifts toward red. At higher concentrations, the primary peak of the fluorescence spectrum becomes a shoulder and the 515.5 nm peak becomes dominant with reduced intensity. This behavior is common to fluorescent organic molecules. At low concentrations, the fluorescence output is low because the number of molecules is low in a unit area of solution. As the concentration increases, the intensity also increases up to an optimal concentration. Beyond this concentration, the fluorescence output intensity starts decreasing due to the proximity of molecules, which suppress the certain singlet vibration and increase the reabsorption. Figure 5b shows a spectral shift of the singlet peak at around 515.5 at higher concentrations, and the shift is due to reabsorption.

The solvent effect was studied in benzene and the trends of the fluorescence intensity and spectral profile were very similar, with a 4 nm redshift in the peak wavelengths at approximately 483 and 508 nm, as displayed in Figure 5c. This shift is due to the change in the solvent dielectric constant.

Figure 5d shows the deconvolution fitting of the PFO-co-PPV-MEHB fluorescence spectral profile using Gaussian functions. The peak positions are 483.3, 508, and 535.4 nm, and the linewidths are approximately 19, 38, and 60 nm, correspondingly. These peaks can be ascribed to the fluorescence counterparts of the singlet oscillators in the simulated UV-VIS spectra. The Stokes shift was calculated in toluene and benzene and it was 98 nm and 97 nm, respectively. The large Stokes shift could be useful to reduce light scattering and self-absorption in optical materials [39].

### 3.3. ASE (Mirrorless Lasing) from CO PFO-co-PPV-MEHB in Toluene

Amplified spontaneous emission occurs due to the stimulated emission and amplification of spontaneously emitted photons under a high population inversion with the single-pass gain of the laser media. ASE without feedback can be considered mirrorless lasing, since most of the features are laser features [40]. Many CPs and COs are capable of producing optically pumped ASE [41,42].

**Figure 6:**
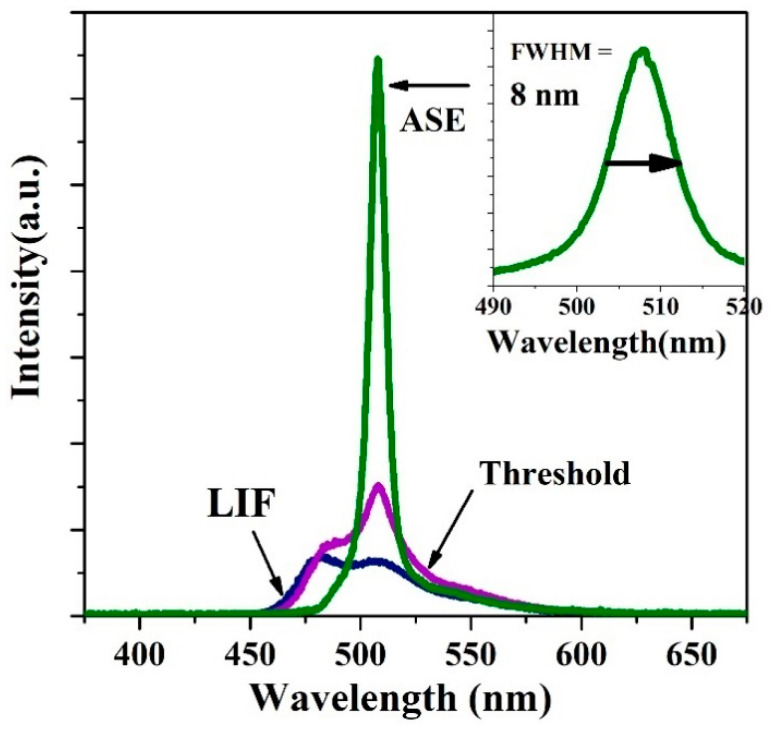
LIF, threshold, and ASE spectra of PFO-co-PPV-MEHB in toluene.

PFO-co-PPV-MEHB in toluene at a concentration of 2.5 mg/mL was transversely excited with a 355 nm Nd: YAG laser. At a minimal pump energy density (3.2 mJ/cm^2^), the laser-induced fluorescence (LIF) was verified and had two peaks at 507 and 481 nm, as shown in Figure 6. After the pump energy density was increased to 4.5 mJ/cm^2^, the spectrum became narrower and peaked at 508 nm with an FWHM of 20 nm. When the pump energy density was 7.75 mJ/cm^2^, ASE was obtained with an FWHM of 8.75 nm and a peak at approximately 508 nm, as presented in Figure 6.

**Figure 7:**
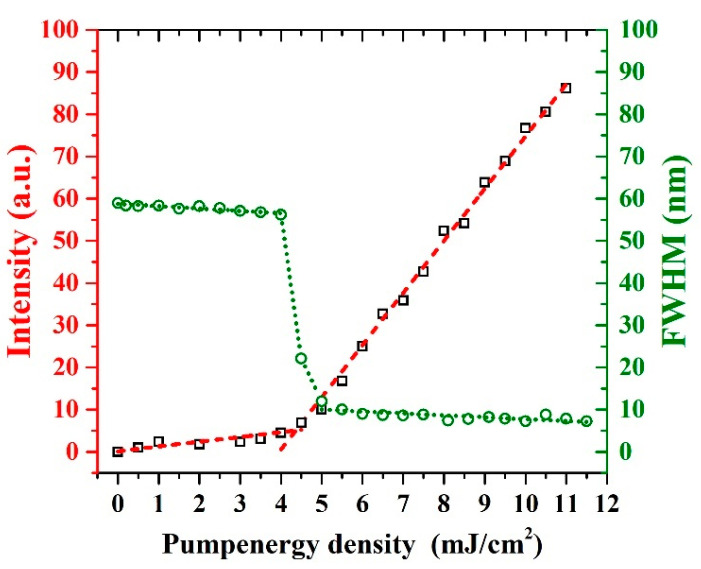
Spectral narrowing and rapid output intensity increase in the CP in toluene as a function of the input pump energy density.

Figure 7 shows the spectral narrowing and increasing of emission intensity of the CP in toluene as a function of the input pump energy density. At 4.5 mJ/cm^2^, the FWHM of the spectrum reduced from 60 to 8.4 nm and the surge of intensity was linear. A additional rise in the input energy density boosted the output strength, however the spectral bandwidth (nm) of ASE was sustained. The reduction in bandwidth (nm) occurred due the net gain maximization near the vibronic transition peaks of the fluorescence spectrum; so, the spectrum displayed intensity surge as the pump energy density raised.

**Figure 8:**
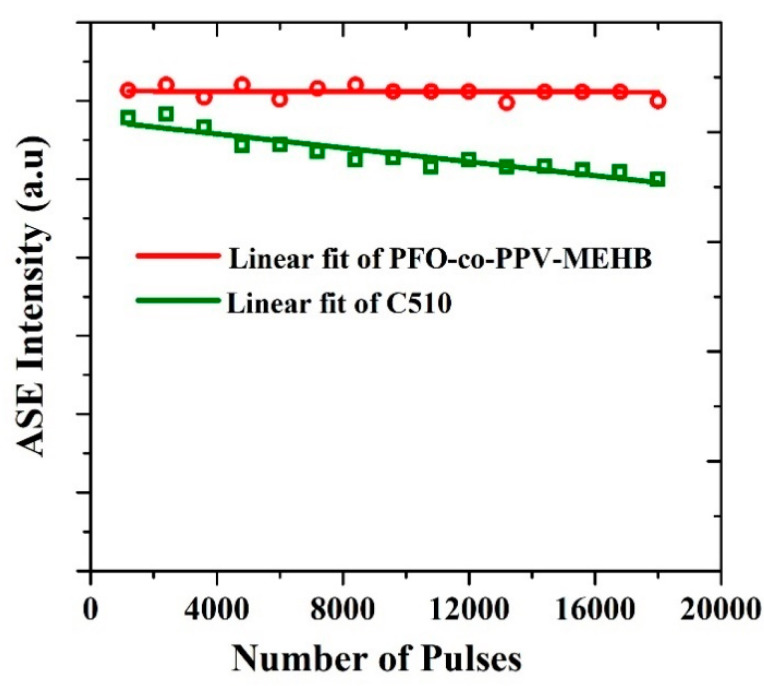
Photochemical stability of the PFO-co-PPV-MEHB ASE in toluene and C510 in methanol at a pump energy density of 12.75 mJ/cm^2^.

Commonly, photostability is estimated by recording the total ASE intensity emitted under a constant pump energy as a function number of pulses or time. The photodegradation is predictable when a decline in the total ASE output intensity is detected [43]. The ASE stabilities of PFO-co-PPV-MEHB (CP) and the laser dye Coumarin 510 (C510) in its best solvent (methanol) were examined and related, as displayed in Figure 8. Both the CP and C510 were kept at a concentration of 2.5 mg/mL, and the pump energy density was 12.75 mJ/cm^2^. The C510 shows signs of degradation, even though the output was high. On the other hand, both the stability and high output of CP did not change much; this shows that CP is a very stable laser material. The CP output was not significantly reduced even after twenty thousand (20 × 10^3^) shots. The CP output was almost maintained, as presented in Figure 8.

## 4. Picosecond Time-Resolved Spectra of ASE in Toluene

**Figure 9:**
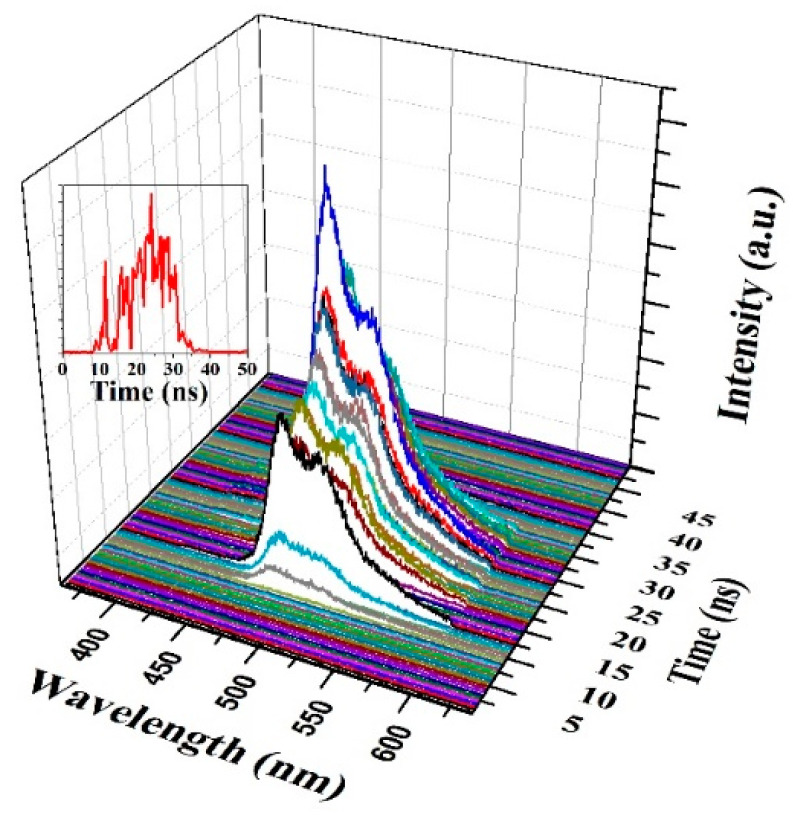
Three-dimensional profiles of LIF, with the inset showing the temporal profile of LIF for a pump energy density of 850 µJ/cm^2^.

We began the TRS studies with a PFO-co-PPV-MEHB solution at a concentration of 2.5 mg/mL and a pump energy density of 850 µJ/cm^2^, as shown in Figure 9. The solution produced only laser-induced fluorescence (LIF) of the CP in the three dimensions when the pump energy density was 3.2 mJ/cm^2^. The CP started fluorescing at 18 ns and maintained this fluorescence until 69.8 ns. The LIF had two peaks at 507 and 481 nm, and the intensity was unstable, fluctuated unsteadily, and reached a maximum value at 48.19 ns.

**Figure 10:**
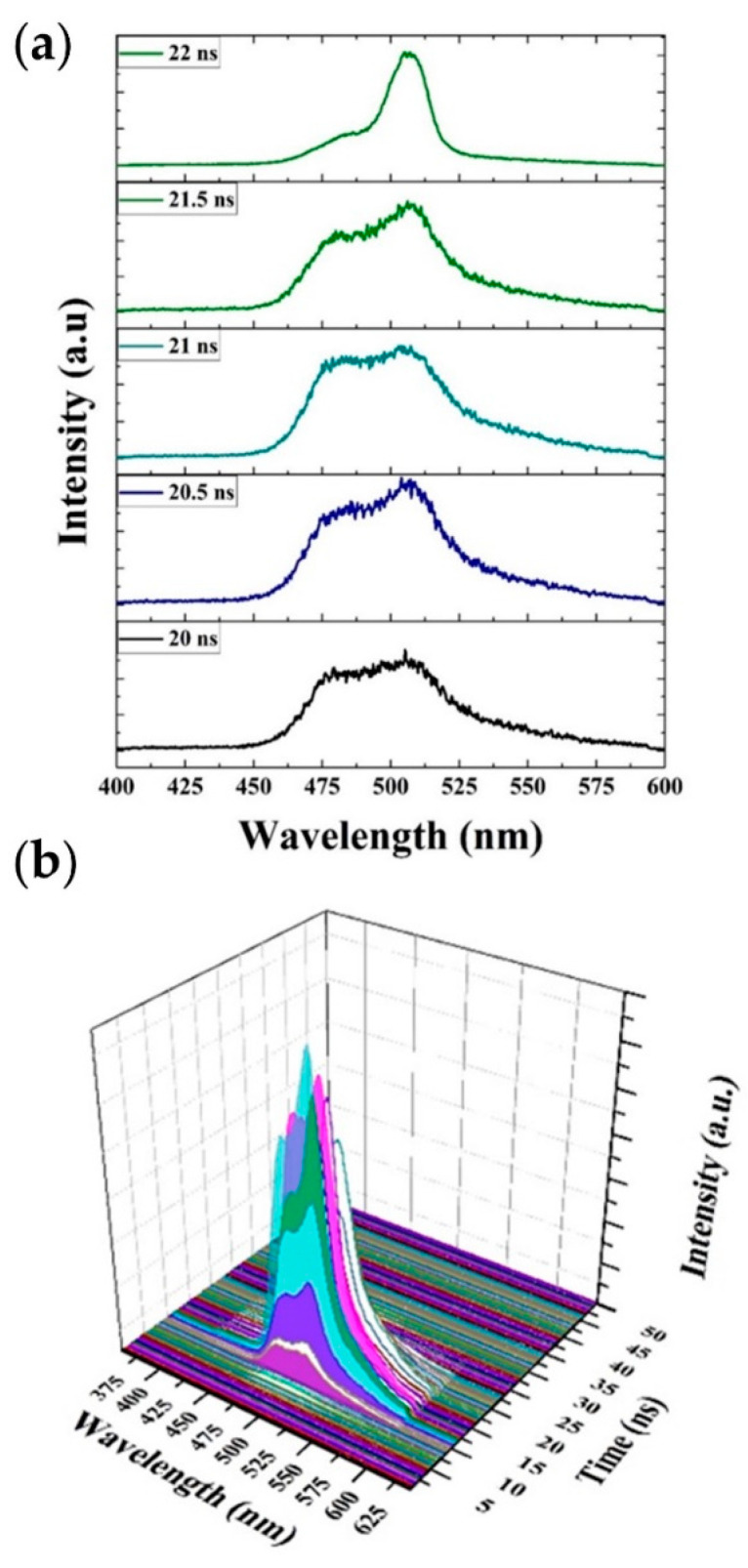
(**a**) Time evolution of the CP threshold spectrum at different time. (**b**) Three-dimensional profiles of the CP solution threshold spectrum for a pump energy density of 4.25 mJ/cm^2^.

Figure 10a shows a few frames of the spectral profile from 20 ns after the Q-switch trigger. The peak acquired was very broad, with an FWHM of 55 nm. As the photon flux increased, the band at approximately 509 nm gained intensity due to the large stimulated emission and single-pass optical gain. The FWHM was 21 nm at 22 ns. Threshold spectral narrowing occurred in 2 ns. The full profile of the spectral narrowing spectra over time is shown in Figure 10b. We can compare Figure 6 with Figure 10; the former gives the transition from fluorescence to ASE as a function of pump energy, while the TRS study shows that the transition from fluorescence to spectral narrowing and the onset of ASE takes 2.5 ns at the same pump energy.

**Figure 11:**
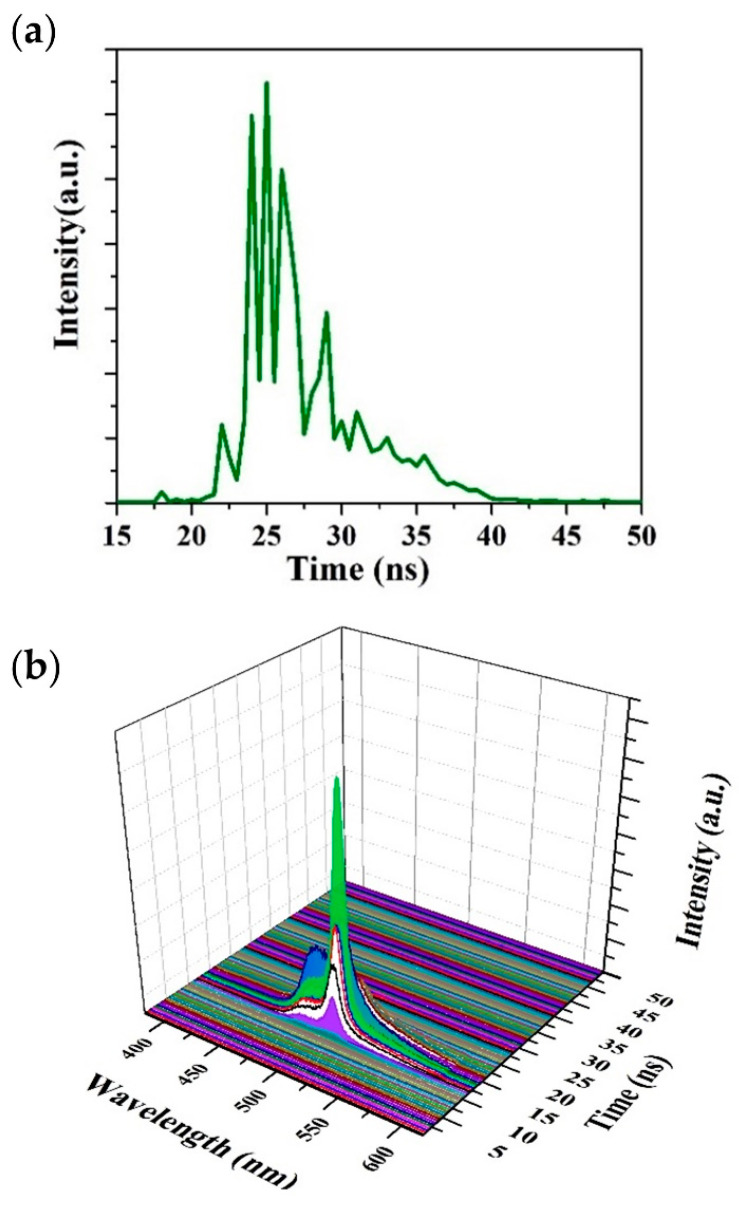
(**a**) Temporal profile of CP ASE for a pump energy density of 7.75 mJ/cm^2^. (**b**) Three-dimensional profiles of CP ASE (1.55 mJ/cm^2^).

The ASE of the CP became intense for pump energy densities above the threshold and was recorded using a picomax spectrometer. Figure 11a shows a Z-slice of the ASE of the CP in toluene at a concentration of 2.5 mg/mL under transverse excitation for a pump energy density of 7.75 mJ/cm^2^. The CP started fluorescing at 24 ns, and the intensity increased with time. The CP produced ASE at 22 ns and reached a maximum intensity at 25 ns. After 25 ns, the ASE intensity decayed up to 34.5 ns and stopped at 35 ns, as displayed in Figure 11a. The ASE peaked at 508 nm with an FWHM of 8 nm, as shown in Figure 11b.

Figure 11b shows the temporal dynamics of the CP in toluene at the aforementioned concentration, but the pump energy density was increased to above 7 mJ/cm^2^. When the pump energy density was 7.75 mJ/cm^2^, ASE was obtained at 520 nm with an FWHM of 8 nm. The entire ASE event became short due to rapid excitation, population inversion, and stimulated emission. The ASE attained the peak in a rapid phase and lasted for only 4 ns. In the pump pulse tailing phase, the photon flux was lower and produced fluorescence at the vibrational band around 480 nm, producing LIF along with weak ASE 2 ns after the maximum ASE peak intensity.

## 5. Conclusions

This report investigated the theoretical, optical, and mirrorless laser properties of CP PFO-co-PPV-MEHB. The calculation showed a HOMO-LUMO structure and a very high oscillator strength of 6.434 (a.u.) for the singlet-singlet transition at 374.43 nm. The experimental UV-Vis spectra obtained for the nonpolar solvent hexane were analogous to the simulated HOMO–LUMO structure. The simulation showed three oscillator strengths that matched the two peaks and shoulder manifested in experimental UV-VIS spectra, indicating that highly efficient fluorescence properties arise from singlet vibrational transitions. Under an optimal solution concentration and pump energy, CP spectral narrowing was achieved at 508 nm with an FWHM of 8.75 nm. The photochemical stability under pulsed laser excitation was excellent, without deuteration for up to 20 k pulses. TRS studies showed a rapid intensity increase and spectral narrowing within 2 ns of fluorescence.

## Data Availability

All data is offered by corresponding author for reasonable request.

## Supplementary Materials

The following are available online at https://www.mdpi.com/2073-4360/13/9/1430/s1. Figure S1: Optimized structure of the copolymer PFO-co-PPV-MEHB Using DFT calculations. Figure S2: (a) Charge distribution of PFO-co-PPV-MEHB (tail-truncated and n = 3 model), (b) Polarizability of PFO-co-PPV-MEHB is 229.15 Å^3^ (tail-truncated and n = 3 model) and (c) H bond donor acceptor of PFO-co-PPV-MEHB (tail-truncated and n = 3 model). Figure S3: Electronic circular dichroism (ECD) of PFO-co-PPV-MEHB (tail-truncated and n = 3 model) calculated using the CAM-B3LYP/6-31G(d,p) basis set. Figure S4 Estimation of HOMO−LUMO gaps for PFO-co-PPV-MEHB using bandgap extrapolation of oligomers (n = 1 to 5).
