# Supplementary material for: TD-DFT Simulation and Experimental Studies of a Mirrorless Lasing of Poly[(9,9-dioctylfluorenyl-2,7-diyl)-co-(1,4-diphenylene-vinylene-2-methoxy-5-{2-ethylhexyloxy}-benzene)]
Source: Polymers (Basel). 2021 Apr 29;13(9):1430. doi: 10.3390/polym13091430 (PMC8125217; doi:10.3390/polym13091430)
Supplement: Supplementary file 1 [file polymers-13-01430-s001.zip › polymers-1158780-supplementary.pdf]

Supplementary Material for:

TD-DFT Simulation and Experimental Studies of a Mirrorless Lasing of Poly[(9,9-Dioctylfluorenyl-2,7-diyl)-co-(1,4-Diphenylene-Vinylene-2-Methoxy-5-{2-Ethylhexyloxy}-Benzene)]

Mamduh J. Aljaafreh [1], Saradh Prasad [1,2], Mohamad S. AlSalhi [1,2,] *, Raya H. Alhandel[1], and Reem A. Alsaigh [1]

[1] Department of Physics and Astronomy, College of Science, King Saud University, Riyadh 11451, Saudi Arabia; srajendra@ksu.edu.sa (S.P.); maljaafreh@ ksu.edu.sa (M.J.A.); rhalhandhal@gmail.com (R.H.A.)) rsaigh@ksu.edu.sa (R.A.A.)

[2] Research Chair on Laser Diagnosis of Cancers, Department of Physics and Astronomy, College of Science, King Saud University, Riyadh 11451, Saudi Arabia

***** Correspondence: malsalhi@ksu.edu.sa; Tel.: +966-50-510-4815.

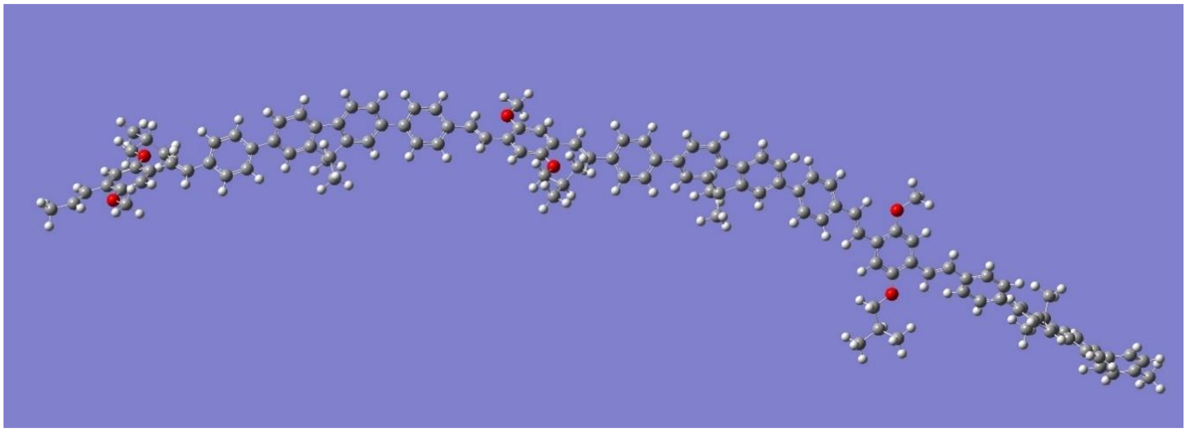

Figure S1 Optimized structure of the copolymer PFO-co-PPV-MEHB Using DFT calculations.

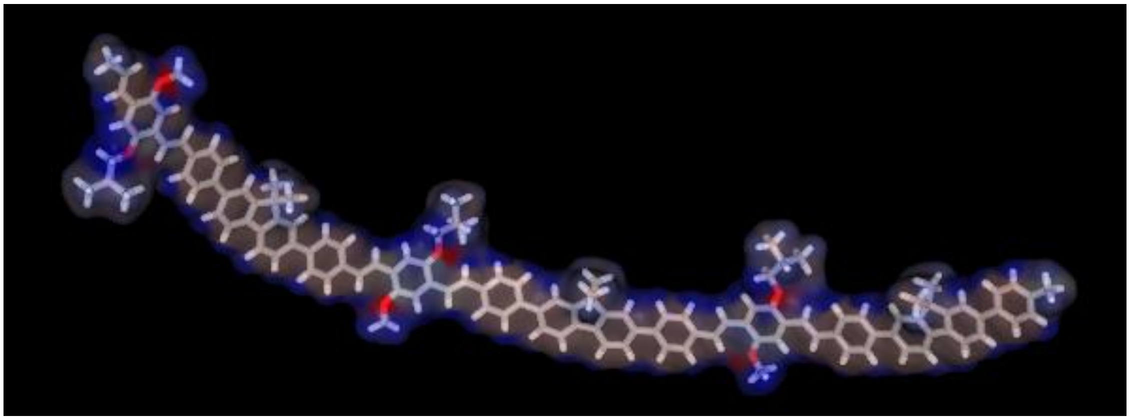

Figure S2 a. Charge distribution of PFO-co-PPV-MEHB (tail-truncated and n= 3 model)

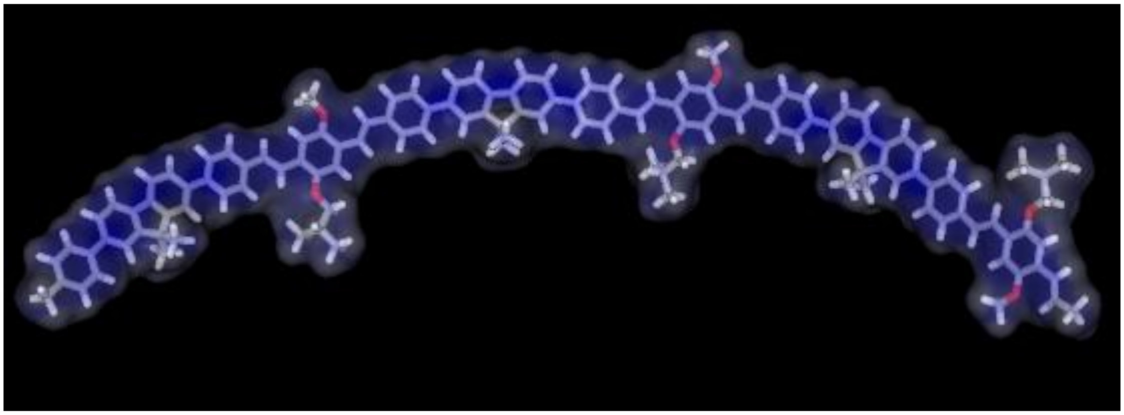

Figure S2 b. Polarizability of PFO-co-PPV-MEHB is 229.15 Å^3 (tail-truncated and n= 3 model)

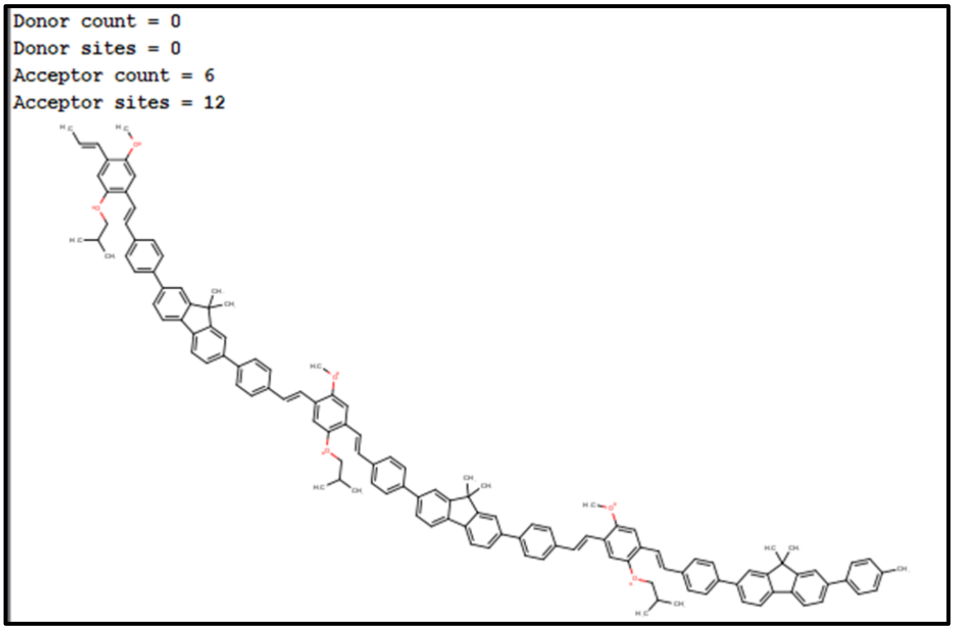

Figure S2 c. H bond donor acceptor of PFO-co-PPV-MEHB (tail-truncated and n= 3 model).

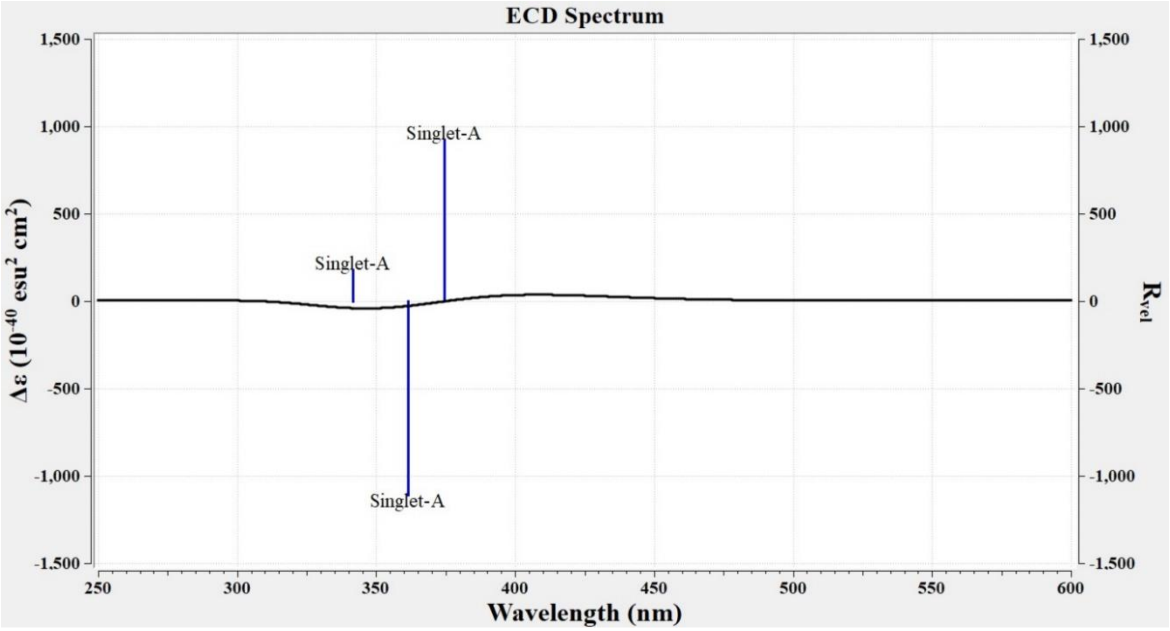

Figure S3. Electronic circular dichroism (ECD) of PFO-co-PPV-MEHB (tail-truncated and n= 3 model) calculated using the CAM-B3LYP/6-31G(d,p) basis set.

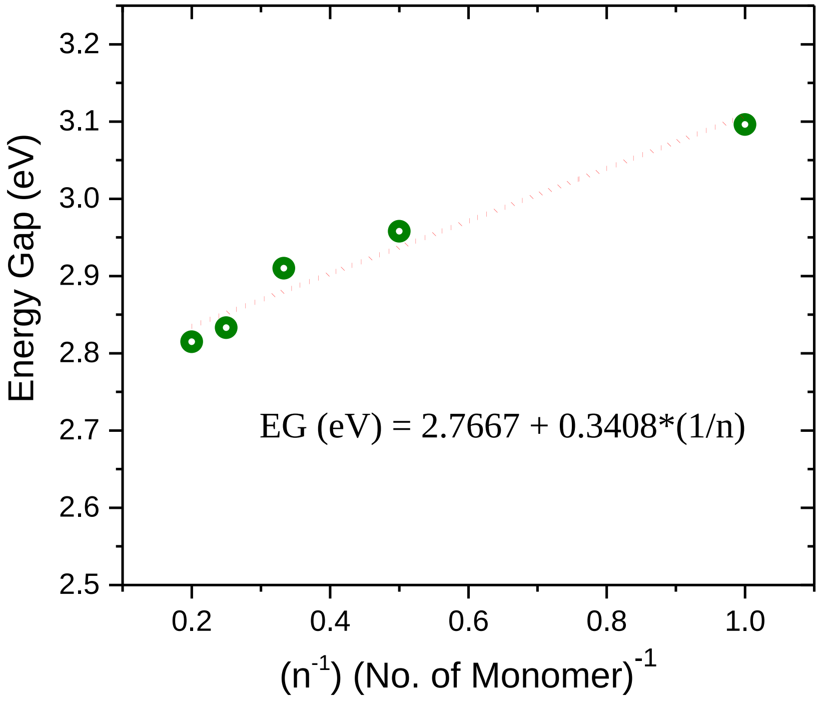

Figure S4 Estimation of HOMO−LUMO gaps for PFO-co-PPV-MEHB using bandgap extrapolation of oligomers (n =1 to 5).
